# When One Stomach Bug Is Not Enough: A Five-Pathogen Gastroenteritis Case Study

**DOI:** 10.7759/cureus.86967

**Published:** 2025-06-29

**Authors:** Erick A Boldt, Emma Newquist, Lily D Rundquist, Daniel Pacciulli, Victoria Echevarria, Tianna Nelson, Azjaah Rogers, Tye Barber

**Affiliations:** 1 Dr. Kiran C. Patel College of Osteopathic Medicine, Nova Southeastern University, Ft. Lauderdale, USA; 2 Dr. Kiran C. Patel College of Osteopathic Medicine, Nova Southeastern University, Clearwater, USA; 3 Family Medicine, Broward Health Medical Center, Ft. Lauderdale, USA

**Keywords:** case report, e. coli, enteroinvasive escherichia coli, enteropathogenic e. coli, foodborne illness, infectious gastroenteritis, norovirus, plesiomonas shigelloides, polymicrobial gastroenteritis, traveler's diarrhea

## Abstract

Foodborne infections are a common cause of gastroenteritis, but cases involving multiple enteric pathogens from a single exposure are rarely reported. We present the case of a healthy 23-year-old male with no significant chronic medical history who developed febrile gastroenteritis after returning from a four-day solo trip to Peru. His symptoms began one day after returning and included fever, fatigue, nausea, vomiting, watery diarrhea, frontal headaches, and severe neck pain. His history was notable for a herniated disc from a motor vehicle collision (MVC) four months prior, for which he was undergoing physical therapy, raising initial concerns for a non-infectious cause of his neck pain. Initial workup revealed leukocytosis, tachycardia, acute kidney injury (AKI), and imaging concerning for a possible small bowel obstruction. Stool polymerase chain reaction (PCR) testing detected multiple enteric pathogens, including *Plesiomonas shigelloides*, enteroaggregative *Escherichia coli* (EAEC), enteropathogenic *E. coli* (EPEC), enteroinvasive *E. coli* (EIEC), and norovirus. The patient was treated with intravenous fluids, ceftriaxone, and metronidazole. His symptoms improved, and he was discharged after two days of supportive care. This case highlights the potential for polymicrobial enteric infections from brief travel-related exposures, emphasizing the need for comprehensive diagnostic testing in returning travelers with severe gastroenteritis.

## Introduction

Acute gastroenteritis (AGE) is a significant global health concern and contributes to an estimated 1.3 million deaths globally per year. In the United States alone, AGE accounts for approximately 179 million cases and one million hospitalizations each year [1]. It typically presents with diarrhea, vomiting, fever, and abdominal pain that lasts for less than a week. While viral pathogens, particularly norovirus, are the most common culprits of epidemic diarrheal disease, bacterial and parasitic co-infections also play a considerable role, especially in foodborne outbreaks and travel-related illnesses [2,3].

A study by Mannstadt et al. examined the prevalence of single versus multiple pathogen infections in gastroenteritis and their association with immunosuppression. They found that approximately 73% of cases involved a single pathogen, while 27% had multiple pathogens, making diagnosis and management more complex [4]. It is hypothesized that multiple pathogen gastroenteritis may occur due to various factors, including impaired host immunity that reduces the body's ability to clear infections, as well as high-risk settings where diverse pathogens circulate, such as international travel or hospitalization [4].

Among the bacterial pathogens mentioned in our patient’s case presentation, enteroaggregative *E. coli *(EAEC) is increasingly recognized as a cause of both acute and chronic watery diarrhea in diverse regions [5]. Enteropathogenic *E. coli *(EPEC) remains a leading cause of diarrheal illness in infants and young children, particularly in resource-limited settings [5]. *Plesiomonas shigelloides*, with antigenic similarities to *Shigella sonnei*, is most often reported in Southeast Asia and Africa, with limited reports from other continents [6,7]. Additionally, enteroinvasive *E. coli* (EIEC) is relatively uncommon due to the requirement of a large inoculum to cause infection [5].

Given the high burden of AGE and its foodborne transmission pathways, understanding the impact of multi-pathogen infections is critical for improving diagnostic accuracy and treatment strategies. This case study explores a complex presentation of AGE in a patient with five enteric pathogens, highlighting the clinical course and management challenges associated with multi-pathogen diarrheal illness and encouraging the necessity for practicing proper food and water safety precautions when traveling.

## Case presentation

In March 2025, a 23-year-old previously healthy male presented to the emergency department with fever, fatigue, and gastrointestinal symptoms. His symptoms began one day after returning from a four-day solo trip to Peru, during which he consumed ceviche, raw shellfish, and unfiltered sink water. He initially developed chills, generalized weakness, and nausea, followed by a single episode of green, large-volume emesis and three episodes of watery, non‑bloody, non‑foul‑smelling diarrhea. He also reported intermittent frontal headaches and worsening severe neck pain, which he described as worse than his usual discomfort from a motor vehicle collision (MVC) four months prior, for which he was undergoing physical therapy. He denied any visual disturbances, photophobia, dysuria, or upper respiratory symptoms.

His medical history was also notable for mild, intermittent asthma. There was no previous history of chronic gastrointestinal disease. He did not take daily medications aside from occasional acetaminophen. There were no reports of tobacco or illicit drug use; however, he did admit to drinking alcohol occasionally. He denied any new sexual partners or history of sexually transmitted infections. Family history was unremarkable for gastrointestinal or infectious diseases.

On arrival, he was febrile (39.5 °C/103.1 °F), tachycardic (HR 121 bpm), and tachypneic (RR 27 bpm), with an elevated blood pressure (158/77 mmHg). He appeared mildly distressed but was alert and oriented. His physical examination revealed dry mucous membranes, no overt abdominal tenderness, and no hepatosplenomegaly. His lung and cardiac exams were unremarkable. Given his fever, headache, and neck pain, a neurological exam was performed, which showed full strength, no focal deficits, and negative Kernig and Brudzinski signs, making meningitis less likely.

**Figure 1:**
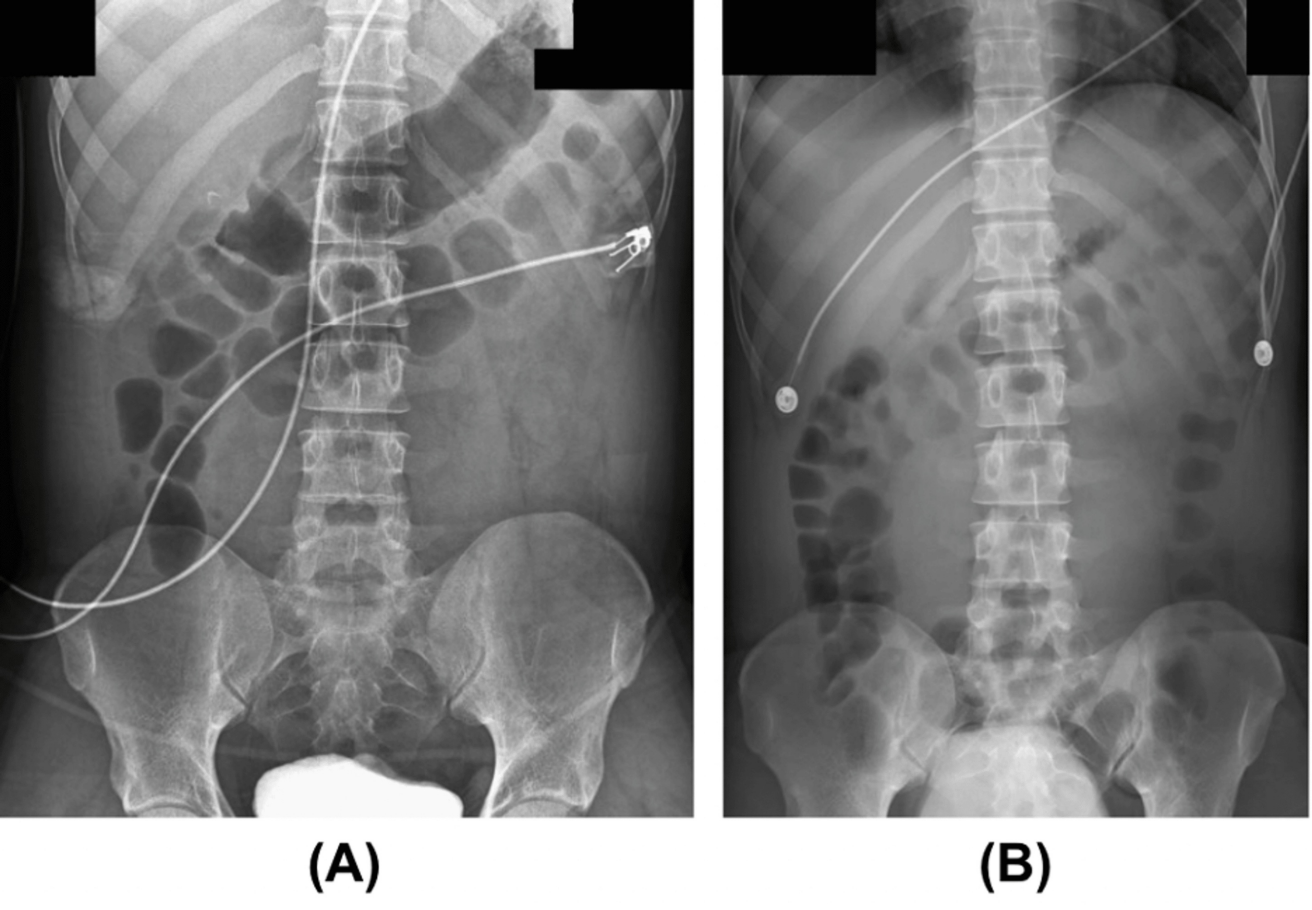
Abdominal X-rays demonstrating evolution of suspected small bowel obstruction (SBO). (A) An abdominal X-ray on the day of admission was significant for a possible SBO due to the presence of dilated small bowel. (B) A repeat abdominal X-ray on hospital day two without significant small bowel dilation signified that the patient did not have an SBO during this admission.

Initial laboratory workup showed a leukocytosis of 12,140 cells per µL of blood (normal rage: 4,000-11,000 cells per µL of blood), lactate of 4.8 mmol/L (normal range: 0.5-2.2 mmol/L), acute kidney injury (AKI) with a creatinine level of 1.4 mg/dL (normal range: 0.8-1.2 mg/dL), and an abdominal radiograph suggested possible small bowel obstruction (SBO) with recommendations to repeat the following morning (Figure 1A). A stool polymerase chain reaction (PCR) panel (Table 1) later revealed multiple enteric pathogens, including *Plesiomonas shigelloides*, EAEC, EPEC, EIEC, and norovirus.

**Table 1:** Results of the gastrointestinal pathogen panel in a 23-year-old male with febrile gastroenteritis following recent travel to Peru. The panel detected multiple enteric pathogens, including *Plesiomonas shigelloides*, enteropathogenic *Escherichia coli *(EPEC), enteroaggregative *E. coli *(EAEC), enteroinvasive *E. coli* (EIEC), and norovirus. Note: Some organisms were categorized into bacterial, viral, and parasitic groups for clarity and conciseness.

Gastrointestinal Panel	Results
Plesiomonas shigelloides	Detected
Salmonella	Not detected
Vibrio PCR, Vibrio cholerae	Not detected
Campylobacter PCR	Not detected
Yersinia eneterocolitica	Not detected
EAEC	Detected
EPEC	Detected
ETEC	Not detected
Shiga-like-toxin producing E. coli (STEC)	Not detected
E. coli O157	Not detected
Shigella/EIEC	Detected
Cryptosporidium, Giardia Lamblia, Cyclospora Cayetanensis, Entamoeba histolytica	Not detected
Adenovirus F 40/41, Astrovirus, Rotavirus, Sapovirus PCR	Not detected
Norovirus	Detected

Given his septic presentation, empiric broad‑spectrum coverage with intravenous ceftriaxone (1 g in sodium chloride 0.9% 50 mL, every 24 hours) and metronidazole (500 mg, every 12 hours) was initiated within the first hour in accordance with our emergency department sepsis protocol and the Infectious Diseases Society of America (IDSA) guidelines for presumed community‑acquired intra‑abdominal infection; therapy was discontinued once stool PCR and blood cultures excluded invasive bacterial pathogens. Since the patient was immunocompetent and without comorbidities that could negatively impact his recovery, the patient’s symptoms were treated with fluid resuscitation and conservative management. Additionally, all positively identified microbes were self-limiting, rendering further medical management unnecessary. A repeat abdominal x-ray on day two was negative for SBO (Figure 1B). On hospital day three, he remained afebrile, tolerated oral intake, and had resolution of nausea and headache, though he continued to have mild non-bloody diarrhea. He was discharged home in stable condition with instructions for continued hydration and monitoring for worsening symptoms.

## Discussion

Though travel-related gastroenteritis is usually attributed to a single pathogen, this case highlights the risk for a polymicrobial enteric infection while traveling to endemic regions. The labs and cultures obtained from our patient were significant for *P. shigelloides*, EAEC, EPEC, EIEC, and norovirus, indicating that there was extensive pathogen exposure that originated from contaminated food or water.

During his four-day trip to Peru, our patient said to have consumed ceviche, raw shellfish, and unfiltered sink water. *P. shigelloides* is a waterborne pathogen associated with consumption of raw seafood and untreated water, both of which were part of the patient’s dietary history [6]. The pathogens EAEC and EPEC are both well-documented causes of traveler’s diarrhea. The co-detection of these further supports a foodborne etiology, and both may have been acquired from contaminated water. Moreover, EIEC is a relatively uncommon cause of enteric infection and requires higher infectious doses to be symptomatic, suggesting the patient was exposed to a substantial microbial load [8]. Finally, norovirus is a highly contagious viral pathogen. Its detection adds to the complexity of his clinical presentation as it can exacerbate symptoms and may contribute to prolonged illness [9]. Overall, the identification of these five distinct pathogens emphasizes the severe and unintended repercussions of consuming unfiltered water and ingesting raw or undercooked foods while traveling abroad, particularly outside the United States.

Retrospective studies support the rarity of this finding. For example, Mannstadt et al. analyzed gastrointestinal PCR panels and found that while 27% of patients had multiple pathogens, fewer than 3% had more than three, and most of those patients were immunocompromised [4]. In contrast, our patient was young and healthy and had no history of chronic illness or immunodeficiency. His exposure history likely contributed to a high inoculum of enteric pathogens, resulting in a rare, compounded infection.

In addition, this case further points out the necessity of a comprehensive workup for travel-related enteric infections. As demonstrated in our patient, a thorough diagnostic approach, including PCR testing, was essential to accurately identify pathogens to guide appropriate management. Healthcare practitioners should maintain a high index of suspicion for polymicrobial infections in patients with a history of recent travel with severe or prolonged gastroenteritis, as having multiple infections can potentially complicate diagnosis and treatment.

Furthermore, our patient’s presentation is particularly apparent given that the patient was a young and healthy individual with no underlying medical conditions or immunocompromising factors. Despite his competent immune system, he still contracted multiple enteric infections on a short four-day trip. It is important to recognize that anyone, regardless of their overall health status, can be susceptible to contracting enteric infections when exposed to contaminated food or water. Though immunocompromised individuals may face more severe outcomes, even those with fully functioning immune systems are still at risk for foodborne illnesses and severe polymicrobial infections. Fortunately, in this case, the patient required only supportive care, highlighting the resilience of a healthy immune system in overcoming such infections.

## Conclusions

This case demonstrates the high possibility for severe polymicrobial gastroenteritis following short-term international travel despite immunocompetency. It also highlights the need for heightened caution in food selection and hygiene practices. Given the patient’s history and clinical presentation, the presence of five distinct enteric pathogens was most likely contracted from consumption of raw seafood and unfiltered water despite his young age and healthy immune status. Healthcare providers should ensure that travelers are well informed about preventive measures, including avoiding raw or undercooked foods, drinking only safe water sources, and practicing good hand hygiene. Our patient’s clinical presentation is a reminder that, while travel can offer enriching experiences, it also comes with inherent risks. Maintaining appropriate pre-travel education and post-travel workup can help reduce the risk of severe polymicrobial gastrointestinal infections, ultimately leading to improved outcomes for individuals experiencing travel-related illnesses.
